# Experiences of mental health and poverty in high-income countries during COVID-19: A systematic review and meta-aggregation

**DOI:** 10.1371/journal.pmen.0000059

**Published:** 2024-10-21

**Authors:** Jessica Allen, Tracy Smith-Carrier, Victoria Smye, Rebecca Gewurtz, Roxanne Isard, Rebecca Goldszmidt, Carrie Anne Marshall

**Affiliations:** 1 Social Justice in Mental Health Research Lab, School of Occupational Therapy, Western University in London, London, Ontario, Canada; 2 School of Humanitarian Studies, Royal Roads University in Victoria, Victoria, British Columbia, Canada; 3 Arthur Labatt Family School of Nursing, Western University in London, London, Ontario, Canada; 4 School of Rehabilitation Science, McMaster University in Hamilton, London, Ontario, Canada; 5 Research & Scholarly Communication Librarian at Western University in London, London, Ontario, Canada; University of North Carolina at Chapel Hill, UNITED STATES OF AMERICA

## Abstract

Systematic reviews have been published that explore the experiences of living in poverty, yet there are no known studies that have synthesized the findings of research exploring the experiences of mental health and wellbeing of persons living in poverty during COVID-19. To address this gap, we conducted a systematic review and meta-aggregation of qualitative evidence using the method described by the Joanna Briggs Institute (JBI) following the PRISMA guidelines. Of 8391 titles and abstracts screened, we included 23 studies in our review and meta-aggregation. In conducting our meta-aggregation, we generated three synthesized findings: 1) magnification of inequities and marginalization during COVID-19; 2) difficulty accessing resources during the lockdown; and 3) the lockdown causing changes in mental health and wellbeing. The findings of this review suggest that persons living in poverty experienced increased difficulties with mental health and well-being during COVID-19. This was largely influenced by the presence of pandemic restrictions and increasing financial precarity that resulted in rising levels of psychosocial distress. Research regarding the plight of persons living in low income is needed to inform policy and practice for future pandemics in order to decrease the vulnerability of this population. Implementing evidence-informed policies and practices that mitigate the negative psychological effects of physical distancing restrictions on persons living in poverty are needed, and these can be identified through future research efforts.

## Introduction

According to the United Nations (UN), the effects of the COVID-19 pandemic has reversed much of the progress made in reducing poverty globally [1]. The pandemic, compounded with other environmental threats to stymie progress on climate change, contributed to an increase of approximately 120 million to the global poor in 2020 [1]. Statistics Canada recognizes that poverty is a complex phenomenon than can only be solved by addressing the factors that cause it beyond income alone (e.g. access to education, health services, employment opportunities, food security, and safe housing) [2]. In high-income countries, individuals or families living in poverty are more likely to experience negative effects from the pandemic due to direct and systematic discrimination that limits equal access to resources of any kind [3]. It also is important to recognize the difference in terms of experience between individuals living in low- and middle-income countries versus those who reside in high-income countries. When investigating issues surrounding experiences of poverty and mental health, the key difference is that high-income countries have greater access to the resources needed to improve upon or resolve these issues. Experiences of low income in high-income countries may be distinctly different than those encountered in low- and middle-income countries, and for this reason they need to be attended to and researched in separate, but effective ways. This difference is often characterized as ‘absolute poverty’ when describing experiences in low-income countries, and ‘relative poverty’ when describing experiences in high-income countries. Absolute poverty refers to the inability to have one’s basic needs met and living in conditions of severe deprivation, whereas relative poverty occurs when an individual’s income is much smaller than the income standard in their society, leading to social exclusion [4].

The resulting psychological stress from COVID-19 was only seen to increase with the implementation of stay-at-home orders, social isolation procedures, closure of facilities, mask mandates, and other protective measures as the pandemic worsened across the world. Beyond the physical toll of COVID-19 and the government-directed closures and restrictions, these factors also influence the mental health and wellbeing of individuals by interrupting meaningful daily life routines and activities [5]. Recently, there has been increasing recognition that mental health issues form the greatest public health challenge of our time, and that the financially impoverished bear the greatest burden of mental illness [6]. According to the Centers for Disease Control and Prevention (CDC) in the US, mental health encompasses an individual’s emotional, psychological, and social well-being and is known to affect how we think, feel, act, and handle stress within the body [7]. A person’s mental health can change over time and is affected by many factors such as available resources, coping abilities, stress levels, childhood experiences, biological factors, drug and alcohol use, and more [7].

Mental health and mental disorders are not the same, although a mental disorder can greatly impact an individual’s overall mental health [7]. A mental disorder is categorized by the World Health Organization (WHO) as a clinically significant change or disturbance to an individual’s behaviour, cognition, or emotional regulation [8]. Although many live “well” with mental disorders, mental disorders are also associated with distress or impairment in important areas of functioning and present themselves in many different forms such as anxiety disorders, depression, bi-polar disorder, post-traumatic stress disorder (PTSD), schizophrenia, eating disorders, addiction, alcoholism, and more [8]. For the purposes of this study, interest will be placed on both general mental health of individuals experiencing poverty as well as the experiences of mental disorders during the COVID-19 pandemic.

Individuals experiencing both poverty and mental health complications should be of interest for research on the effects of COVID-19 as it has been established that this population is at a higher risk of experiencing negative impacts from the pandemic [9]. There is an urgent need for research on this topic, as knowledge will contribute to understanding the factors influencing poor mental health among those living in poverty during Covid-19, as well as contribute to the development of practice and policy solutions based on the experiences described by this population. This knowledge is essential for promoting positive social change as well as supports for better mental health among this population.

### Research on experiences of poverty and mental health during COVID-19

According to a study by Benfer et al., the COVID-19 pandemic has magnified longstanding social and health inequities among persons who live in poverty [3]. Given their vulnerability, persons living in poverty are of interest when exploring the ways in which the pandemic and the resulting social policies influence experiences of mental health and well-being. Further research in this area will provide guidance during future pandemics, as well as instruction on how to navigate social isolation methods with fewer adverse consequences on mental health and well-being. While qualitative research describing the mental health experiences of persons living in poverty have been generated for many years [10, 11], few studies have explored the impacts of COVID-19 on persons living in low income, and there are no known systematic reviews that have synthesized this literature to date. Providing a clear and comprehensive overview of the existing literature and evidence on this topic is essential for informing both future research and relevant policymakers concerning poverty, mental health, and COVID-19.

### The current study

While there are many studies that explore the topics of COVID-19, mental health, and poverty separately, there are no known studies that have synthesized the findings of qualitative research exploring the mental well-being of persons living in poverty during COVID-19. We conducted this systematic review and meta-aggregation to fill this gap in the existing literature. Specifically, in this study we aim to: 1) describe the existing qualitative literature focused on mental health experiences among individuals living in poverty during COVID-19; 2) aggregate the findings of these studies; and 3) describe the quality of research that exists on this topic.

## Methodology

We conducted a systematic review and meta-aggregation of qualitative evidence following the guidelines for systematic reviews provided by the Joanna Briggs Institute (JBI) to explore experiences of mental health and well-being of individuals living in poverty during the COVID-19 pandemic [12]. This review was prospectively registered with PROSPERO on July 27, 2023 (Registration #CRD42023448207).

### Search strategy

A search strategy was developed in collaboration with an Academic Research Librarian, an author on this study (RI). We deployed this search in July 2023 where we searched the following six electronic databases: Medline; EMBASE; CINHAL; PsychINFO; Nursing & Allied Health Database; and Sociological Abstracts. Three concepts guided the development of this strategy, with key terms selected pertaining to the concept of mental health (i.e. well-being, mental disorder), poverty (i.e. financial hardship, income insecurity) and COVID-19 (i.e. pandemic, health emergency). These three sets of key terms were combined using a Boolean ‘AND’ to identify relevant studies. In addition to this search, a hand search of the reference lists of all included studies was completed to identify any additional research not captured using the search strategy. See S1 Appendix for samples of the database searches.

### Study selection

**Fig 1:**
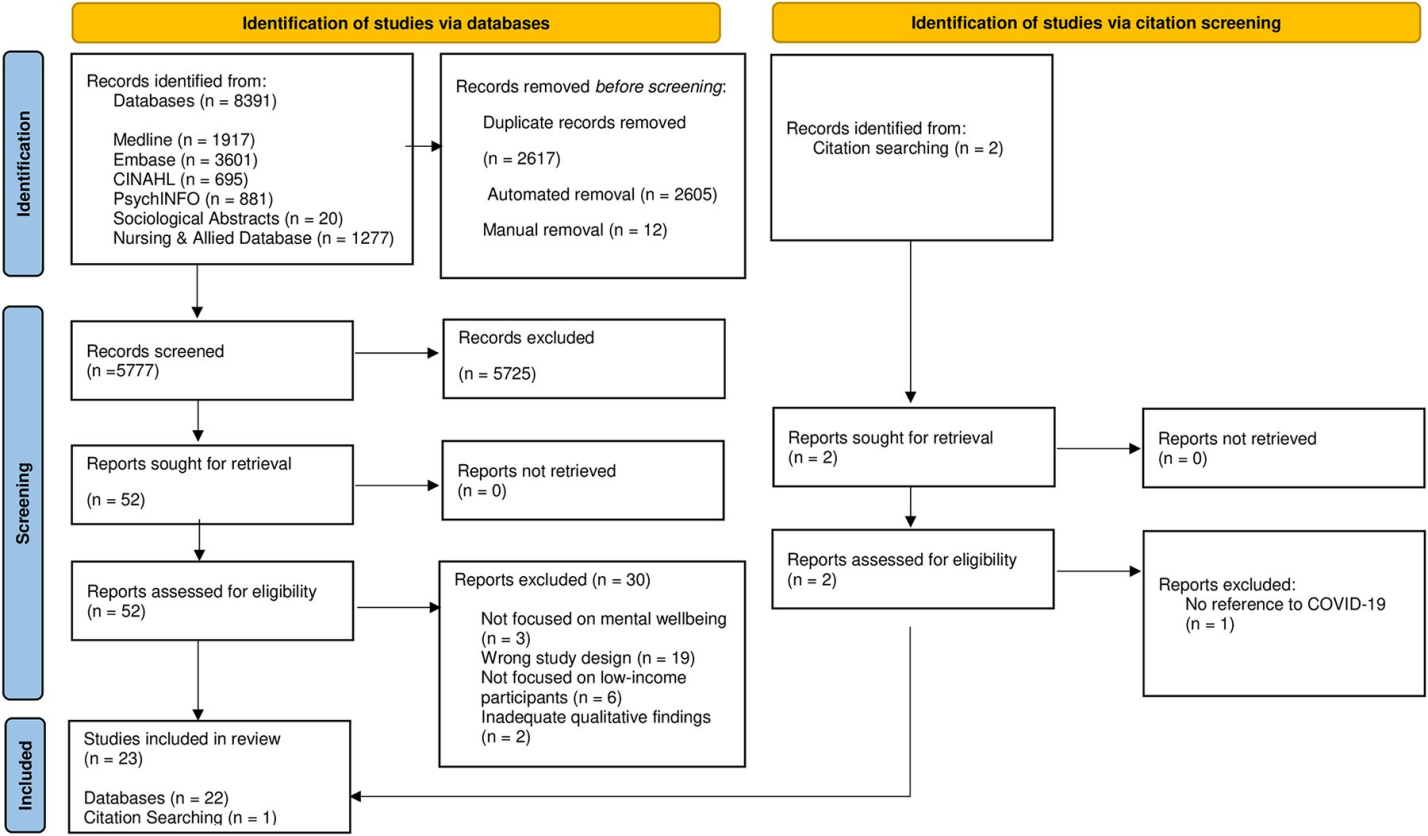
PRISMA flow diagram.

Two independent raters (JA and RG) conducted a title and abstract screening and full text review using Covidence, a cloud-based systematic review software program [13]. At both the title and abstract and full-text review stages, two independent raters used inclusion and exclusion criteria to guide the selection of studies. These criteria are detailed in Fig 1. Conflicts emerging at the title and abstract and full-text review phases were resolved through discussion and consensus among the two raters.

### Data extraction

Using a custom data extraction form developed in Covidence [13], we extracted the following information from the included studies: sample country; year of publication; study design; methodology; sample size; gender; race/ethnicity; sexual orientation; clinical and population characteristics; and journal discipline.

### Critical appraisal

We conducted a critical appraisal using the JBI Critical Appraisal Checklist for Qualitative Studies [14]. We assigned a score of 1 to each rating of “yes” and 0 to each rating of “unclear” and “no” for each of the 10 criteria. Each included study was assigned a score ranging from 1–10, and any study that was given a score of less than 5/10 was excluded from the meta-aggregation. This ensured that only moderate to high quality studies would be included and employed to guide and inform future research and policies.

### Meta-aggregation

Using the method described by the JBI, we conducted a meta-aggregation of the themes extracted from the included studies [12]. Meta-aggregation is a process that seeks to synthesize the findings of a range of qualitative studies to advance the development of future research and inform policymakers and practitioners [12]. In conducting this meta-aggregation, we selected themes from included studies that responded to the research question describing the experiences reflecting the mental health of individuals living in low income during COVID-19. Primary themes were identified from included studies; however, if a secondary theme was deemed more relevant to the topic of mental health and poverty during COVID-19, then only the sub-theme was included within the meta-aggregation.

First, we extracted themes from included studies that responded to the research question posed. For improved organization, we extracted each theme name along with a brief description into a table in Microsoft Word. Time was spent reading and re-reading the theme descriptions to best understand the experiences being presented by participants, and how these experiences may have overlapped or differed between individuals. We then worked to organize the included themes into clusters or groups based on similarities to create appropriate categories for the collected themes. As suggested by Lockwood et al., [12] categories of data were then refined further and grouped together. Over-arching descriptions of these groups of categories formed synthesized findings that represent the themes presented from the included studies. Two members of our research team (CM and JA) discussed and refined the synthesized findings, and we incorporated feedback provided at this time into the meta-aggregation.

## Findings

**Fig 2:**
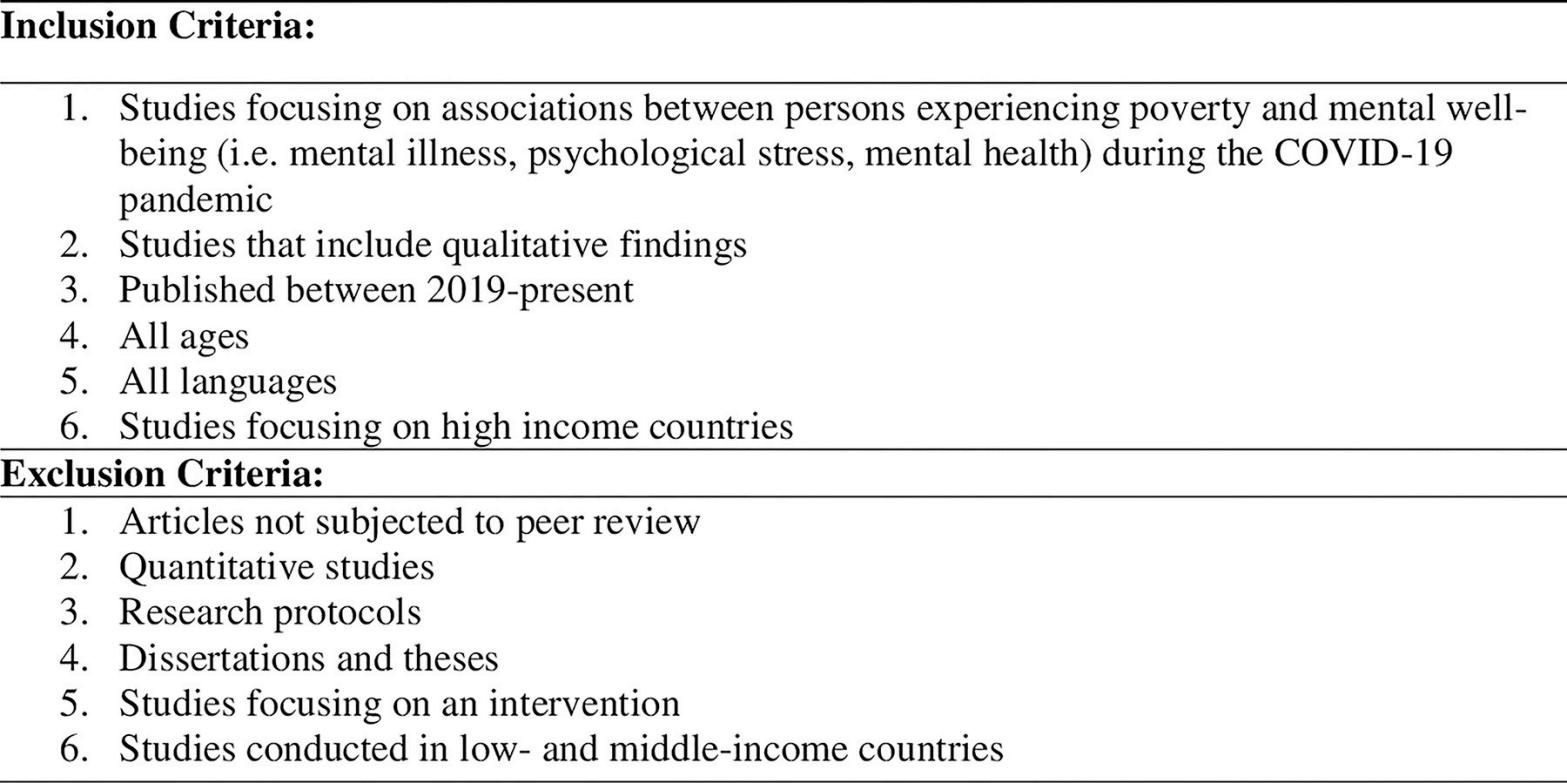
Inclusion and exclusion criteria.

The search yielded a total of 8391 title and abstracts from the six databases, of which 2614 were removed as duplicates. 5723 records were removed at the title and abstract screening stage, leaving 52 studies for full-text review. Upon searching the reference lists of the included studies, two additional studies were identified that fit the eligibility criteria. The resulting 54 studies were screened at the full-text level. A total of 23 articles met the criteria for inclusion. A more detailed account of this process can be found within the PRISMA flow diagram provided in Fig 2.

### Participant characteristics

We included 23 studies representing n = 789 participants. Participants consisted of persons living in low-income located in high-income countries, most of whom lived with a diagnosed mental illness or have reported poor mental health or well-being. Participants included n = 505 women (64.0%), n = 152 men (19.3%), and n = 7 other genders (0.9%). The included studies derived mostly from the United States (n = 11 studies; 47.8%), followed by Canada (n = 3 studies, 13.0%), the UK (n = 2 studies, 8.7%), and New Zealand (n = 2 studies, 8.7%). Many participants described themselves as Hispanic/LatinX (n = 184, 23.3%), White (n = 108, 13.7%), or Black (n = 93, 11.8%). See Table 1 for a summary of the demographic characteristics of participants, Table 2 for a summary of the combined characteristics of included studies, and Table 3 for a description of the characteristics of individual studies.

**Table 1:** Demographic characteristics of participants in included studies.

Characteristic	
Participant Characteristics (n = 789)	n (%)
Gender	
Women	505 (64.0)
Men	152 (19.3)
Other genders	7 (0.9)
Not specified	125 (15.8)
Race/Ethnicity ^a^^,^ ^b^	
Hispanic/LatinX	184 (23.3)
White	108 (13.7)
Black	93 (11.8)
Asian	67 (8.5)
Indigenous	27 (3.4)
Mixed race/ethnicity	22 (2.8)
Other	2 (0.3)
Unknown/Not specified	295 (37.4)
Sexual Orientation ^b^	
Heterosexual	76 (9.6)
2SLGBTQ+	18 (2.3)
Questioning	3 (0.4)
Other/Unspecified	692 (87.0)
Clinical Characteristics ^a^^,^ ^b^	
Mental Health Condition	120 (15.2%)
Physical Disability or Health Condition	82 (10.4%)
Cognitive Health Condition	17 (2.2%)
Two or More Health Conditions	27 (3.4%)
No Health Condition (mental, physical, cognitive)	86 (10.9%)
Unspecified	622 (78.8%)
	n Studies (%)
Participant Population	
Mothers	5 (21.7)
College/University	2 (8.7)
Youth Experiencing Homelessness	2 (8.7)
Older Adults	2 (8.7)
Refugees/Migrants	1 (4.3)
Women	1 (4.3)
Varied	10 (43.5)
Sample Country	
USA	11 (47.8)
Canada	3 (13.0)
New Zealand	2 (8.7)
UK	2 (8.7)
Germany	1 (4.3)
Ireland	1 (4.3)
Peru	1 (4.3)
Netherlands	1 (4.3)
Israel	1 (4.3)

**Note:** Percentage sums do not all equal 100 due to rounding

^a^ Participant frequencies for race/ethnicity and clinical characteristics exceed the total number of participants due to the classification of participants in more than one category in the included studies

^b^ Due to discrepancies in reporting across the individual studies, the number of participants identified in each of these categories should be treated as estimates

**Table 2:** Combined characteristics of included studies.

Characteristic	
Year of Publication	n Studies (%)
2021	9 (39.1)
2022	8 (34.8)
2023	6 (26.1)
Study Design	
Qualitative	17 (73.9)
Mixed methods	6 (26.1)
Methodology	
General Qualitative	11 (47.8)
Mixed methods	6 (26.1)
Phenomenology	5 (21.7)
Participatory (including photovoice)	1 (4.3)
Journal Discipline	
Interdisciplinary	18 (78.3)
Psychiatry	1 (4.3)
Psychology	1 (4.3)
Occupational therapy	1 (4.3)
Medicine	1 (4.3)
Veterinary	1 (4.3)

**Note:** Percentage sums do not all equal 100 due to rounding

**Table 3:** Characteristics of included studies.

Authors	Methodology	Discipline	Sample Country	Sample Size	Sample Characteristics	JBI Critical Appraisal Score (range 1–10)	Study Design
Choi, Giridharan, Cartmell, Lum, Signal, Puloka, Crossin, Gray, Davies, Baker & Kvalsvig (2021)	General Qualitative	Medicine	New Zealand	n = 27	Varied	9	Qualitative
Curtin, O’Shea, & Hayes (2022)	General Qualitative	Interdisciplinary	Ireland	n = 15	Mothers	7	Mixed Methods
Davis, Lu, Williams, Roas-Gomez, Leziak, Jackson, Feinglass, & Yee (2022)	General Qualitative	Interdisciplinary	USA	n = 40	Mothers	8	Qualitative
Duh-Leong, Yin, Yi, Chen, Mui, Perrin, Zhao, & Gross (2022)	General Qualitative	Interdisciplinary	USA	n = 25	Mothers	8	Qualitative
Elers, Jayan, Elers, & Dutta (2021)	General Qualitative	Interdisciplinary	New Zealand	n = 35	Varied	7	Qualitative
Flores-Flores, Otero-Oyague, Rey-Evangelista, Zevallos-Morales, Ramos-Bonilla, Carrión, Patiño, Pollard, Parodi, Hurst, Gallo, & Reynolds (2023)	General Qualitative	Interdisciplinary	Peru	n = 40	Older adults	10	Qualitative
Higashi, Sood, Conrado, Shahan, Leonard, & Pruitt (2022)	General Qualitative	Interdisciplinary	USA	n = 40	Varied	9	Qualitative
Horner, Hugh-Jones, Sutherland, Brennan, & Sadler-Smith (2022)	Phenomenology	Interdisciplinary	United Kingdom	n = 20	University Students	8	Qualitative
Jumaa, Bendau, Ströhle, Heinz, Betzler, & Petzold (2023)	General Qualitative	Psychiatry	Germany	n = 10	Refugees/Migrants	7	Mixed Methods
Kiebler & Stewart (2021)	Photovoice	Interdisciplinary	USA	n = 28	University Students	7	Qualitative
Krumer-Nevo, & Refaeli (2021)	General Qualitative	Psychology	Israel	n = 123	Varied	9	Mixed Methods
Marshall, Gewurtz, Holmes, Phillips, Aryobi, & Smith-Carrier (2023)	General Qualitative	Occupational Therapy	Canada	n = 27	Varied	10	Mixed Methods
May, Aughterson, Fancourt & Burton (2023)	General Qualitative	Interdisciplinary	United Kingdom	n = 20	Varied	7	Qualitative
Morris, Wu, & Morales (2021)	Phenomenology	Veterinary	Canada	n = 12	Varied	7	Qualitative
Noble, Owens, Thulien, & Suleiman (2022)	General Qualitative	Interdisciplinary	Canada	n = 45	Youth	10	Qualitative
Perrigo, Samek, & Hurlburt (2022)	General Qualitative	Interdisciplinary	USA	n = 31	Varied	7	Qualitative
Quandt, LaMonto, Mora, Talton, Laurienti, & Arcury (2021)	General Qualitative	Interdisciplinary	USA	n = 105	Mothers	7	Mixed Methods
Radey, Lowe, Langenderfer-Magruder, & Posada (2022)	General Qualitative	Interdisciplinary	USA	n = 34	Mothers	9	Qualitative
Reber, Kreschmer, DeShong, & Meade (2021)	General Qualitative	Interdisciplinary	USA	n = 15	Varied	10	Qualitative
Rew, Yeargain, Peretz, & Croce (2021)	Phenomenology	Interdisciplinary	USA	n = 20	Youth	9	Qualitative
Schmitt, Dimong, Maroko, Phillips-Howard, Gruer, Berry, Nash, Kochhar, & Sommer (2023)	General Qualitative	Interdisciplinary	USA	n = 25	Women	9	Qualitative
van der Kamp, Torensma, Vader, Pijpker, den Broeder, Fransen, & Wagemakers (2023)	Phenomenology	Interdisciplinary	Netherlands	n = 37	Varied	8	Qualitative
Winship, Gendron, Waters, Chung, Battle, Cisewski, Gregory, Sargent, Zanjani, Slattum, Mackiewicz, Diallo, Ford, Falls, Price, & Parsons (2021)	Phenomenology	Interdisciplinary	USA	n = 15	Older Adults	8	Mixed Methods

### Critical appraisal

Critical appraisal scores ranged from 7–10 (Mdn = 8; IQR = 2) on the JBI Critical Appraisal Checklist for Qualitative Studies [14]. This indicates that the methodological quality of included articles was moderate to high; individual scores can be found in Table 3.

## Meta-aggregation

We extracted 73 themes from all 23 studies. These themes were then clustered into 10 categories, and 3 synthesized findings. A summary of each of the themes, categories, and synthesized findings is provided on Table 4.

**Table 4:** Meta-aggregation of themes.

Study	Theme	Category	Synthesized Finding
Davis et al., (2022)** **	New barriers to providing for their children (p. 900–901)	Effects of the lockdown magnifying financial stress (9)*	Magnification of inequities and marginalization during COVID-19 (26)*
Higashi et al., (2022)** **	The pandemic increased economic hardship (p. 1031–1032)		
Kiebler & Stewart (2022)** **	Changed environmental demands (p. 211–213)		
Krumer-Nevo & Refaeli (2021)** **	Economic difficulties (p. 427)		
May et al., (2023)	Stress and worry caused by lack of employment (p. 6)		
Morris et al., (2021)** **	The emotional impact of compounding barriers related to accessing veterinary care (p. 4)		
Morris et al., (2021)** **	The barriers to accessing veterinary care pre- and peri-COVID-19 (p. 3)		
Radey et al., (2022)** **	Navigating resource-limited networks (p. 4–5)		
Rew et al., (2021)	Financial instability (p. 654–655)		
Duh-Leong et al., (2022)** **	Heightened family hardship (p. 51)	Forced to take increased risks in the contexts of poverty (6)*	
Elers et al. (2021)** **	Everyday living (p. 111)		
Higashi et al., (2022)** **	The pandemic increased psychological distress (p. 1033)		
Horner et al., (2022)** **	Conflict of health vs. wealth (p. 58–59)		
Marshall et al., (2023)** **	Limitations on time use entrenching inequities (p.144-146)		
Reber et al., (2022)** **	Contexts where fear, isolation, and invisibility were experienced (p. 6–7*)*		
Horner et al., (2022)** **	The challenge of liminality (p. 57–58)	Struggling to cope or access resources during the lockdown (5)*	
May et al., (2023)** **	Emotional and practical support from friends, family, and the community (p. 4)		
May et al., (2023)** **	Shame and stigma around asking for help (p. 7)		
Schmitt et al., (2023)** **	Heightened experiences of menstrual related anxiety and shame (p. 6–7)		
Schmitt et al., (2023)** **	The adoption of coping strategies in response to menstrual product insecurity (p. 5–6)		
Elers et al., (2021)** **	Communicative inequality (p. 111–112)	Ignored needs and further marginalization in society (6)*	
Kiebler & Stewart (2022)** **	Comparison with others (p. 213–215)		
Marshall et al., (2023)** **	Covid has magnified the inequities I live with (p. 146–147)		
Marshall et al., (2023)** **	Classist and ableist pandemic policies have made daily life so much harder (p. 147)		
Quandt et al., (2021)** **	Latinx community attitudes (p. 39–40)		
Reber et al., (2022)** **	Feelings and emotional reactions (p. 4–5)		
Choi et al., (2021)** **	Coping strategies (p.61)	Strategies used to reduce worsening mental health and financial precarity (13)*	Difficulty accessing resources during the lockdown (24)*
Duh-Leong et al., (2022)** **	“We care more about the baby. We can just eat whatever food, it’s okay” (p. 53–54)		
Flores-Flores et al., (2023)** **	Self-regulation of emotion and stress (p. 1112–1113)		
Higashi et al., (2022)** **	The pandemic increased food needs, leading to greater food insecurity (p. 1032–1033)		
Jumaa et al., (2023)** **	Belief in God and other protective strategies (p. 68)		
Jumaa et al., (2023)** **	Avoidance of an Arab neighborhood in Berlin (p.69)		
Krumer-Nevo & Refaeli (2021)** **	Successful personal coping (p. 428)		
May et al., (2023)** **	Strategies to ease financial difficulties resulted in poorer physical and mental health (p. 6–7)		
Noble et al., (2022)** **	Increased substance use as a coping mechanism (p. 10)		
Radey et al., (2022)** **	Reassessing network member relationships (p. 5–6)		
Radey et al., (2022)** **	Establishing new boundaries for in-person contact (p. 6–7)		
Rew et al., (2021)** **	Maladaptive coping (p. 655–656)		
Schmitt et al., (2023)	Financial and physical barriers to menstrual product access (p. 4–5)		
Choi et al., (2021)** **	Experience of lockdown (p. 58–61)		
Curtin et al., (2022)** **	Impact on parents (p. 9–12)	Social isolation caused by the lockdown (6)*	
Elers et al., (2021)** **	Relational and community agency (p. 112–113)		
Horner et al., (2022)** **	Trapped with too much time and too many thoughts (p. 55–57)		
Marshall et al., (2023)** **	Feeling imprisoned in one’s home (p. 146)		
Noble et al., (2022)** **	Isolation and loneliness (p. 8)		
Curtin et al., (2022)** **	Impact on children (p. 5–7)	Fears regarding the socioemotional well-being of children during the lockdown (5)*	
Curtin et al., (2022)** **	Loss of services (p. 7–9)		
Davis et al., (2022)** **	Challenges of parenting multiple children (p. 898–899)		
Duh-Leong et al., (2022)** **	“Because he’s too young to wear a mask” (p. 51, 53)		
Perrigo et al., (2022)** **	Poor parent and child well-being (p. 5)		
Curtin et al., (2022)** **	Positive impact on families (p. 7)	Positive effects of the lockdown on wellbeing and relationships (8)*	The lockdown causing changes in mental health and well-being (23)
Flores-Flores et al., (2023)** **	Maintenance and restoration of social bonds and relationships with family and friends. (p. 1113–1114)		
Krumer-Nevo & Refaeli (2021)** **	Personal and family improvement (p. 428)		
Marshall et al., (2023)** **	Finding meaning through relationship with activity (p. 146)		
Marshall et al., (2023)** **	Striving for meaning (p. 146)		
Morris et al., (2021)** **	The human-animal bond and resilience in the context of the COVID-19 pandemic (p. 4)		
Perrigo et al., (2022)** **	Pandemic-related benefits (p. 5)		
Radey et al., (2022)	Discovering emotionally available networks (p. 4)		
Jumaa et al., (2023)** **	Mental health issues due to COVID-19 (p. 65–68)		
Krumer-Nevo & Refaeli (2021)** **	Personal and family difficulties (p. 427–428)	Pre-existing mental health conditions exacerbated by effects of the lockdown (6)*	
May et al., (2023)** **	Increased loneliness due to reduction in social activities to save money (p. 7)		
Noble et al., (2022)** **	Mental health challenges (p. 8–9)		
Rew et al., (2021)** **	Mental health (p. 655)		
van der Kamp et al., (2023)	Psychological impact (p. 5–6)		
Choi et al., (2021)** **	Impact of the outbreak on people’s lives (p. 58)		
Davis et al., (2022)** **	Barriers to maintaining self-care (p. 899–900)	Emotional stress caused by fear of COVID-19 and its effects (9)*	
Kiebler & Stewart (2022)** **	Family and personal health vulnerabilities (p. 215)		
Quandt et al., (2021)** **	Work (p. 35–37)		
Quandt et al., (2021)** **	Overall concerns (p. 41–42)		
Rew et al., (2021)** **	Relationship conflict and loss (p. 655)		
van der Kamp et al., (2023)** **	Social unity and divide (p. 6–7)		
Winship et al., (2022)** **	Individual context (p. 7–8)		
Winship et al., (2022)** **	Polarized views of COVID-19 precautions (p. 6)		

* Total number of extracted themes included within the category or synthesized finding

Synthesized findings included: 1) Magnification of inequities and marginalization during COVID-19; 2) Experiencing difficulty when accessing resources during the lockdown; and 3) The lockdown causing changes in mental health and well-being. Each of these synthesized findings and their corresponding categories are described in detail below.

### Magnification of inequities and marginalization during COVID-19

A total of 26 (36%) themes identified the ways in which COVID-19 caused participants living in low-income to experience increased feelings of marginalization within society. Ignored needs and amplified financial stress led study participants to feel a sense of “othering” between themselves and individuals with higher incomes. These themes were extracted from 15 of the included studies (65.2%) and are presented as three categories generated within the meta-aggregation: 1) Effects of the lockdown magnifying financial stress; 2) Forced to take increased risks in the contexts of poverty; 3) Struggling to cope or access resources during the lockdown; and 4) Ignored needs and further marginalization in society.

#### Effects of the lockdown magnifying financial stress

Nine themes identified in eight studies demonstrated how low-income individuals experienced an increase in financial stress and worry during COVID-19. Many studies indicated that factors such as reduced hours or job loss due to the pandemic were root causes of psychological stress and anxiety [15–19]. The resultant financial precarity left many participants with inadequate income to afford necessities to survive such as food or rent [15, 18, 20, 21]. Two studies addressed participants having to make difficult choices of where to spend their limited financial resources as they could not manage to pay for rent, food, and healthcare all at once [15, 22]. Increased financial pressures and uncertainty during COVID-19 caused participants to experience feelings such as significant stress, excessive worrying, anxiety, suffocation, and poor well-being [17, 18, 20–22]. Financial stress was still present in participants receiving government assistance or grants, as the funds provided were still insufficient to cover all of participants’ daily necessities. [15, 20]. Uncertainty and worry about the future were commonly reported as participants described the rapidly-changing nature of the pandemic and its negative implications on low-income earners [16, 17, 20].

#### Forced to take increased risks in the contexts of poverty

There were six themes identified in six different studies that pertain to participants’ experiences of increased risk during COVID-19. In each of these studies, living in poverty was identified as a factor that caused increased conflict for individuals concerning the risk of contracting the virus. In several cases, fears about leaving the home to work conflicted with worries about not having enough financial resources to survive [15, 23–25]. This internal conflict seemed to be heightened for participants who worked in high-risk/‘essential’ jobs or those who lived with young children or older adults, as they feared spreading the virus to these more vulnerable populations [15, 25]. Two studies described how being in low-income engendered higher risks for people seeking to navigate access to basic needs such as groceries [26, 27]. Participants described how those with higher incomes could afford the luxury of grocery delivery while remaining in the safety of their home; whereas individuals living on a lower-income were forced to take more risks during COVID-19 to access basic needs such as food [26, 27]. While grappling with this conflict, the majority of participants reported increased concern or psychological distress during this time.

#### Struggling to cope or access resources during the lockdown

Five themes were identified in three different studies that described how participants struggled to cope financially or access important resources during COVID-19, exemplifying the inequities experienced by this population. These three studies highlight how participants experienced negative feelings such as guilt, shame, or failure when they were unable to afford the necessities for a health life [17, 21, 28]. For example, individuals who identified as low-income students reported a reduction in employment and student wages during the lockdown, causing a financial reliance on family members [23]. These experiences promoted feelings of failure in this population as it was difficult for participants to see higher-income students easily affording to live [23]. In the article by May et al. [17], individuals living in poverty described experiencing feelings of shame and embarrassment when disclosing their financial precarity to friends and family members. Asking for financial help from loved ones and relying on charitable resources such as the food bank were reported by participants to promote feelings of social stigma and belittlement [17]. The study by Schmitt et al. [28], reflects the experiences and barriers for low-income women accessing menstrual products and resources during COVID-19. Participants described being unable to afford menstrual products, which promoted negative mental health implications such as low self-esteem, embarrassment, and depression. These individuals recounted how they felt uncomfortable in their own homes due to insufficient access to menstrual protection, which resulted in intense feelings of shame [28].

#### Ignored needs and further marginalization in society

Six themes identified in five studies explored the ways in which low-income individuals perceived or experienced stigma and marginalization during COVID-19. Many participants expressed frustration or distrust with the government and other social systems such as health care [25–27]. Specifically, participants questioned how these systems continue to operate while marginalizing a large group of already-vulnerable people such as those living in poverty [27]. Individuals narrated accounts of being unable to successfully navigate these systems due to issues such as high costs, language barriers, and decreased mobility [16, 25, 26]. Low-income Canadians felt like their specific needs did not matter to their government due to the fact that the Canadian Emergency Response Benefits (CERB) provided over the pandemic was offered at a much higher amount that other monthly income support payments such as those receiving social assistance, unemployment or disability grants [27]. Interviews in another study showed that low-income students perceived high-income students as being able to easily afford and navigate educational systems and resources such as mental health help [16]. Other examples of marginalization described in this population include how low-income Latinx individuals perceived an increase in racism, such as being blamed for COVID-19 or even being refused services owing to their racial and socioeconomic status [29]. Participants living with a disability identified a sense of being forgotten during the lockdown and expressed that their needs were unimportant to the government, leading to a belief that society that does not care about them has humans [26]. Feelings of fear and isolation were reported, as living with a disability was deemed to already limit their mobility within society [26]. Participants expressed how living in this state could easily ‘push’ an individual into depression or a poor state of mental health [26].

### Difficulty accessing resources during the lockdown

A total of 25 themes from 17 (74%) studies explored the reduction or loss of access to resources and services during COVID-19 that are necessary in promoting positive health and well-being for low-income individuals. Participants recounted how they coped physically and emotionally during the pandemic with minimal resources at their disposal. These themes are presented as three categories: 1) Strategies used to reduce worsening mental health and financial precarity; 2) Social isolation caused by the lockdown; and 3) Fears regarding the socioemotional well-being of children during the lockdown.

#### Strategies used to reduce the worsening of mental health and financial precarity

Thirteen themes identified from 11 studies explored the ways in which participants coped with worsening financial precarity and mental health during COVID-19. Participants created ways to navigate life during isolation while experiencing limited access to important resources. In two of the articles, families reported having to develop strategies to adequately provide meals in lieu of material hardship [15, 24]. These methods included stockpiling child essentials such as diapers and formula, swapping ingredients in meals for cheaper, less-nutritious options, and borrowing small amounts of money where possible from the people around them [15, 24]. Other participants described attempting to offset food expenses by skipping meals altogether as the concept of building inescapable debt caused feelings of being trapped and psychological pain [17]. Participants identifying as women reported buying menstrual products in bulk to reduce anxiety and cope with possible future product shortages [28].

Individuals also described the strategies they used to achieve comfort during this time of isolation and uncertainty. The most common strategy used to reduce mental stress among low-income individuals was distraction. Finding things to occupy one’s time such as exercise, cleaning, cooking, or hobbies such as painting or baking were seen as successful ways to deal with unwanted emotions during COVID-19 [21, 30–32]. According to some participants, activities that required learning and personal development were most successful in promoting positive well-being among low-income individuals [21]. Two studies recorded that religion or a belief in God was helpful in coping with uncertainty due to COVID-19 as such beliefs or practices could help ease stressful thoughts [31, 32]. In the study by Radey et al [19], mothers found that ending relationships with individuals who did not respect the COVID-19 safety guidelines was a strategy that reduced their stress around contracting the virus.

Participants also recounted ways of coping with poor mental health that were not as successful or healthy. Three studies reported an increase of drug and alcohol use as coping mechanisms during COVID-19 for individuals living in poverty [17, 18, 33]. Without access to other activities due to the lockdown, boredom and heightened mental health concerns caused some participants to rely on negative behaviours such as binge drinking and/or increased substance use [18, 33].

#### Social isolation caused by the lockdown

Six themes identified in six studies explore how social isolation due to COVID-19 made it difficult for low-income individuals to access important resources, services, and social connections that promote positive well-being and mental health. Almost all participants reported feelings of stress, isolation, and loneliness during the various periods of lockdown over the course of the pandemic [17, 23, 30, 33, 34]. Isolation during this time caused many individuals to live without a sense of social connection to their comminutes or family members, resulting in feelings of disconnect and poor mental health [23, 30]. Others reported that the lack of access to one’s usual coping mechanisms or social resources that promoting positive well-being contributed to feelings of distress and a sense of being forgotten [23, 33]. Healthcare organizations that, due to isolation policies, banned patients from bringing support people to healthcare appointments acted as a barrier to health resources as this may have exacerbated medical anxieties and language barriers for some patients [25]. Overall limitations on social activity during COVID-19 impacted the way low-income individuals were able to navigate and have access to important resources for well-being.

#### Fears regarding the socioemotional well-being of children during the lockdown

Five themes identified in four studies explored feelings of distress for low-income parents due to limited access to vital resources for their children during periods of isolation. School was seen as an important social resource that children could not access during COVID-19. Due to school closures, children were denied access to daily social connection and engagement which caused concerns for parents regarding social skill regression, and worsening behaviours such as ‘meltdowns’ [34]. Participants recounted how children often expressed feelings of boredom, frustration, and anxiety resulting from the lockdown measures [20, 35]. Parents also spoke to their own challenges regarding lacking the skills and resources needed to help their children succeed in online school activities, which caused overwhelming feelings for some participants, especially those with multiple children [20]. The study by Duh-Leong et al. [24] describes how accessing healthcare with an infant during COVID-19 can be challenging as wearing protective measures can be difficult for such young people. Parents with infants also described being unable to ensure the level of cleanliness or safety of their child within settings such as the clinic, eliciting feelings of increased stress [24]. Participants from another study described difficulty accessing important medical services and appointments during COVID-19 [34]. These parents expressed concerns regarding their child(ren)’s development and milestone achievements due to cancelled appointments with medical professionals such as speech therapists or psychologists [34].

### The lockdown causing changes in mental health and well-being

A total of 23 themes identified from n = 17 (74%) studies explored the ways in which isolation due to COVID-19 caused changes in mental health and well-being for low-income individuals. Participants expressed how COVID-19 has affected their personal well-being in both positive and negative ways. Three categories were generated that characterize this synthesized finding: 1) Positive effects of the lockdown on well-being and relationships; 2) Pre-existing mental health conditions exacerbated by effects of the lockdown; and 3) Emotional stress caused by fear of COVID-19 and its effects.

#### Positive effects of the lockdown on well-being and relationships

Eight themes identified in seven studies explored how COVID-19 created positive changes in aspects of well-being for low-income individuals. It was commonly discussed among participants that increased time spent with family had a positive effect on their well-being [19, 29, 34, 35]. Due to work and school closures, participants found they had more time to spend with their children, resulting, at times, in a strengthened relationship between child and parent [19, 34]. Older adults often used the extra time with family to engage in meaningful activities such as babysitting grandchildren or helping with family chores which were reported to increase confidence and self-esteem among this population [31]. Some families described the benefit of being able to live a slower lifestyle without being rushed due to extra time during isolation [35]. With fewer opportunities to engage in meaningful activities, some participants expressed increased drive and motivation to find meaning in activities that they could participate in or did not have time for previously [27]. A few participants shared that a positive effect of the lockdown was being able to teach children new concepts while they were at home, such as mutual care for family members [21]. Narratives from one study specifically capture the positive effect of time spent with pets in promoting resilience and well-being in their low-income owners during times of isolation [22]. Although COVID-19 restrictions may have disrupted the lives of this population, individuals living in low-income report experiencing some positive effects on their well-being due to the prolonged periods of isolation.

#### Pre-existing mental health conditions exacerbated by effects of the lockdown

There were six themes identified from six studies that explored how COVID-19 had caused pre-existing mental health concerns to worsen for low-income individuals. Existing psychological distress was reported as worsening due to COVID-19, shutting down many daily occupations and promoting fear regarding family members in other countries where the healthcare systems were deemed not strong [32]. Distress caused by work changes and home care were also reported to contribute to an exacerbation of pre-existing psychological conditions [36]. According to a study by van der Kamp et al. [36], when compared to higher-income individuals, low-income respondents experienced a more intense impact of isolation causing pre-existing psychological conditions to become more prominent. In addition, low-income participants reported experiences of increased depression, anxiety, PTSD, anger, desperation, and fear during the isolation period [21]. Specifically, conditions such as PTSD and anxiety were exacerbated during this time as aspects of the pandemic mimicked situations of trauma for some [33]. In a study focused on youth experiencing homelessness, it was reported that pre-existing mental health conditions were aggravated by COVID-19, including depression and anxiety disorders [18]. According to another study, financial and social strain were factors that led to the exacerbation of mental health conditions [17].

#### Emotional stress caused by fear of COVID-19 and its effects

Nine themes from seven studies explore how COVID-19 caused emotional stress and fear resulting in changes in the mental health of low-income individuals. According to three studies, a common source of emotional stress reported at this time was non-compliance with safety restrictions and implementations [29, 36, 37]. Many participants reported feelings of frustration and distress when others would not use personal protective equipment (PPE) [36, 37]. Emotional stress was also reported by participants to be a result of worry about the health of their loved ones and family members [16, 29, 30, 37]. This was seen to especially be true for individuals with family members who were at high risk or vulnerable to contracting the virus [16, 29]. The mental health and emotional well-being of participants changed during COVID-19 as fear around infection, transmission, and essential service interactions grew with the severity of the virus [30]. In a study by Davis et al. [20], mothers described how changes in sleep, overthinking, and anxiety were caused by stress resulting from the COVID-19 restrictions. Participants from one study reported on how the deaths of people they knew made the lockdown even more difficult to handle emotionally, causing changes to their well-being during this time [18].

## Discussion

We conducted this systematic review to synthesize the findings of the qualitative literature exploring the mental health and well-being experiences of individuals living in poverty during COVID-19. In this research we identified 23 qualitative and mixed methods studies that represent the mental health experiences of individuals living in low-income during COVID-19. This meta-aggregation revealed that mental health and well-being were challenged during the pandemic, especially in times of isolation or lockdown. Both individual and structural factors contributed to overall psychological distress and changes in well-being among individuals living on a low-income during this time. Individual factors such as stress related to caring for children or living with the symptoms of a pre-existing mental illness were exacerbated during the pandemic and added to the psychological stress that participants experienced. The findings of this review also suggest that persons living in poverty experienced mental health and well-being challenges due to financial precarity that increased during COVID-19. Experiences of persons living in low-income differed from the experiences of individuals living on higher-incomes during COVID-19, as mental health and well-being are shaped by an individual’s economic and social environment [6]. Participants in the included studies described the negative impacts of social isolation and loneliness on their own mental health and well-being during COVID-19, a finding that is consistent with other recent literature on this topic [38].

The findings of this review suggest that living in low income during COVID-19 prevented individuals and families from meeting their basic needs such as affording housing, food, and healthcare. Further, participants included within this review reported reduced work hours and job loss during COVID-19 which promoted feelings of significant stress and poor well-being [16, 17, 19–21]. Participants described having to make difficult choices regarding where to spend limited financial resources, often not being able to afford the requirements of a healthy life [15, 18, 20, 21]. This finding is consistent with recent literature pertaining to material hardship and low-income earners as poverty has been shown to contribute to a cascade of negative physical and mental health outcomes later in life [39]. Further, this emphasizes the importance of addressing financial precarity among individuals living in low-income so that persons marginalized by poverty can obtain the resources necessary for survival and to thrive. There is a pressing need to address the high cost of basic needs such as groceries or healthcare to enable those living in poverty to succeed in an increasingly unaffordable society.

Almost half of the included studies in the review were conducted with samples residing in the USA, with only a few studies published in other countries such as Canada, New Zealand, and the UK. Since the start of the pandemic in 2019, research has grown over time with most studies published in 2021 and 2022. Participants in these studies represented a range of races, genders, and clinical characteristics; however, the race/ethnicity of nearly 40%, and sexual orientation of over 85% of participants were unreported. Only one study included in this review focused specifically on the experiences of those living with a moderate to severe disability/impairment [26] however, none focused specifically on racialized or other groups marginalized by inequity. Understanding the unique experiences of specific subgroups is critical in informing social or structural interventions that specially target the mental health needs of these populations during future pandemics.

### Research implications

The findings of this review highlight the importance of understanding the experiences of individuals living in poverty during global disruptions such as the COVID-19 pandemic. Many participants in the included studies expressed distrust and frustration with the existing social systems such as the government and healthcare systems due to increased difficulty accessing and navigating these systems during the pandemic [25–27]. Future research investigating the experiences of individuals living in poverty within specific social contexts, as they interact with specific structural supports and resources is needed. Research should target systems such as income support including, workfare and welfare systems, social and healthcare services, and housing support to further understand the interactions and issues that occur within these systems and to identify avenues that might increase resources and access to supports for persons who are living on a low-income. In doing so, researchers can offer research gleaned from persons living in low-income to policymakers in order to facilitate meaningful social change and encourage practice methods that are evidence-based.

Participants who were parents largely described fears regarding the socioemotional well-being of their children during COVID-19 and the possible lasting effects the pandemic might have on their overall development [20, 34, 35]. Considering this review did not exclude participants under the age of 18, it is surprising that none of the existing studies focused on the perspectives of children living in poverty during COVID-19. This finding suggests that future qualitative research should consider investigating the experiences of future pandemics and how these experiences influence the mental health of children. According to a systematic review of mental health impacts of COVID-19 on children, youth are at crucial phases of development which makes them more susceptible to the negative mental health impacts of the health emergency [40]. Therefore, future research in this area should examine the mental health experiences of youth living in poverty during, and after health emergencies to document how these affect their development, as well as possible interventions to aid this population in future times of significant isolation and/or crisis.

To expand on this literature, researchers conducting studies on the mental health experiences of individuals living in poverty during health emergencies should consider collecting the demographic data on their participants. Information on the gender, race/ethnicity, and sexual orientation of participants could aid in exploring how intersecting social or structural issues that contribute to mental health challenges of specific groups. As identified within this review, there is a need for research that specifically focuses on key sub populations such as people identifying as gender fluid or gender non-conforming or are members of the 2SLGBTQIA+ community, or other racialized groups such as those from indigenous backgrounds. As the samples included in this review largely resided in the USA, there is a need to conduct research on the mental health experiences of individuals living in low-income during COVID-19 in other countries, such as those in the global south.

### Practice implications

The findings of this review reveal that many persons living in low income experienced negative changes in mental health and well-being during the COVID-19 pandemic. This finding is consistent with recent literature that highlights how persons living in poverty bear the greatest burden of mental illness in society due to structural, institutional, and social oppression or disadvantage [6]. Practitioners should consider the multiple barriers that persons experiencing poverty face, with a focus on advocating for better financial resources and eliminating the social stigma as described by participants within this review. It is important for mental health practitioners to ensure that poverty-aware practice is utilized in services through education and training of service providers and other healthcare practitioners. When supporting the well-being of their clients, practitioners should include poverty as a key consideration in their assessments and approaches to care, aligning with a social determinants perspective of health [6]. This review also identifies the need to adapt mental health services to more effectively meet the needs of persons living in poverty during future global emergencies, such as health pandemics or climate disasters, given the lack of access to services that participants described during COVID-19. Possible solutions could lie within the implementation of practices identified in the research literature as effective, as well as an internal reflection and evaluation of these services by practitioners to ensure that all service-users are treated and addressed in a fair/equitable manner.

### Policy implications

In some countries, such as Canada, governments enacted temporary emergency benefits to offset the deleterious impacts of the pandemic [41]. However, in these instances, there was no specific financial support awarded to individuals who were already experiencing financial precarity prior to the onset of COVID-19. Persons receiving social assistance described feelings of increased marginalization as the CERB presented significantly higher financial resources for people shut out of work than individuals living on general and disability-linked social assistance [27]. Therefore federal, regional, and municipal policymakers should consider the financial and psychological impact that the pandemic had on the population of people already on social assistance and work to develop policy proposals that could offset the impact of poverty during future health emergencies or global crises. Shifting social assistance policy by adopting Universal Basic Income (UBI) or Basic Income Guarantee (BIG) would both mitigate the effects of poverty during future pandemics, while also providing incomes for individuals whose employment may be affected through the use of the same funding mechanism. UBI has been identified as one of the most powerful, straightforward solutions to effectively addressing poverty [42]. Recent literature indicates that UBI can improve the financial situation of individuals and families, but also to improve the mental health and well-being of this population by alleviating economic precarity and worry [42]. UBI can also reduce poverty-related stigma as well as promoting social and financial independence for low-income individuals [43]. Alleviating financial precarity and stress by increasing the incomes of persons living in poverty would improve mental health and well-being.

### Limitations

The evidence presented in this systematic review primarily reflects the mental health experiences of individuals living in poverty during the COVID-19 pandemic in the US and the findings should be interpreted as limited to this context. Since this review exclusively investigated the perspectives of those residing in high-income countries, our findings reflect that specific context. Studies that describe the experiences of individuals in low-income countries are needed. Further, the findings of this review primarily reflect the perspectives of women, as a majority of participants in the included studies identified as such, suggesting the need for research on this topic that includes men and other genders. While the demographic characteristics of participants reflected a range of races and ethnicities, there was little data that reflected the perspectives of Indigenous or racialized persons.

## Conclusions

This systematic review highlights the importance of attending to the mental health of individuals living in poverty during a global pandemic, as well as the unique barriers that this population faced during this time. This research emphasizes the need for policies that further increase access to mental health services for persons living in poverty when barriers are imposed by the need for physical distancing. Researchers, practitioners, and policy makers should consider how COVID-19 and it effects deepened mental health inequities among persons living in low income and advocate for fair, resilient systems that can better support persons living in poverty. Evidence-informed approaches, such as UBI, would eliminate poverty and place people who would otherwise be at risk of poverty into in a less vulnerable position before the onset of future pandemics, thereby pre-emptively mitigating mental health inequities. Adaptation of services and policies so that they are specifically tailored to the needs of low-income individuals will help to mitigate the health inequities observed during this historic time.

## Supporting information

S1 Checklist PRISMA checklist. (DOCX)

S1 Appendix Database searches. (DOCX)

S1 File Excluded articles and reasons for exclusion. (DOCX)
