# Supplementary material for: Experiences of mental health and poverty in high-income countries during COVID-19: A systematic review and meta-aggregation
Source: PLOS Ment Health. 2024 Oct 21;1(5):e0000059. doi: 10.1371/journal.pmen.0000059 (PMC12798167; doi:10.1371/journal.pmen.0000059)
Supplement: S1 Appendix — (DOCX) [file pmen.0000059.s002.docx]

Appendix 1

Database: **Ovid MEDLINE**

Search Strategy:

--------------------------------------------------------------------------------

1     poverty.mp. or Poverty/ (71216)

2     economic deprivation.mp. (867)

3     resource scarcity.mp. (596)

4     impoverished.mp. (4156)

5     penniless.mp. (7)

6     inadequate resources.mp. (505)

7     poor living conditions.mp. (386)

8     inadequate living conditions.mp. (26)

9     lack of income.mp. (69)

10     no income.mp. (212)

11     lack of money.mp. (401)

12     no money.mp. (149)

13     unemploy*.mp. (25886)

14     low ses.mp. (3579)

15     Low socioeconomic status.mp. or Low Socioeconomic Status/ (6903)

16     economic insufficiency.mp. (3)

17     bankrupt*.mp. (1481)

18     poor community.mp. (279)

19     poor family.mp. (612)

20     poor individual*.mp. (196)

21     slum*.mp. (4809)

22     low income.mp. (46791)

23     income security.mp. (921)

24     income insecurity.mp. (45)

25     financial hardship.mp. (1281)

26     equity.mp. (30808)

27     health equity.mp. or Health Equity/ (9627)

28     inequity.mp. (6137)

29     inequitable.mp. (2537)

30     1 or 2 or 3 or 4 or 5 or 6 or 7 or 8 or 9 or 10 or 11 or 12 or 13 or 14 or 15 or 16 or 17 or 18 or 19 or 20 or 21 or 22 or 23 or 24 or 25 or 26 or 27 or 28 or 29 (179098)

31     mental health.mp. or Mental Health/ (263073)

32     mental wellness.mp. (319)

33     mental disorder*.mp. (222795)

34     mental stability.mp. (79)

35     mental hygiene.mp. (3275)

36     anxiety disorder.mp. or Anxiety Disorders/ (52660)

37     social anxiety.mp. (7797)

38     Anxiety/ or anxiety.mp. (304149)

39     life satisfaction.mp. (10807)

40     psychological health.mp. (7913)

41     psychological wellbeing.mp. (2498)

42     emotional intelligence.mp. or Emotional Intelligence/ (4458)

43     emotional regulation.mp. or Emotional Regulation/ (4997)

44     psychological resilience.mp. or Resilience, Psychological/ (9025)

45     psychological adaptation.mp. or Adaptation, Psychological/ (104148)

46     coping behaviour.mp. (388)

47     mental fatigue.mp. or Mental Fatigue/ (2876)

48     Depression/ or depress*.mp. (640771)

49     alcoholism.mp. or Alcoholism/ (89780)

50     psychological stress.mp. or Stress, Psychological/ (138049)

51     optimism.mp. or Optimism/ (11224)

52     hopeful*.mp. (15874)

53     social isolation.mp. or Social Isolation/ (24257)

54     social exclusion.mp. (2563)

55     social deprivation.mp. or Social Deprivation/ (2221)

56     mood.mp. (99843)

57     mood disorder.mp. or Mood Disorders/ (20744)

58     affective disorder.mp. (8180)

59     psychotic disorders.mp. or Psychotic Disorders/ (56216)

60     Schizophrenia/ or schizophrenia.mp. (159130)

61     psychosis.mp. (45738)

62     substance-use.mp. (49692)

63     substance-related disorder.mp. or Substance-Related Disorders/ (105060)

64     substance misuse.mp. (3504)

65     addiction.mp. (58521)

66     personality disorders.mp. or Personality Disorders/ (26195)

67     panic disorder.mp. or Panic Disorder/ (11845)

68     stress-related disorders.mp. (1960)

69     trauma-related disorders.mp. (403)

70     PTSD.mp. (32976)

71     post-traumatic stress disorder.mp. or Stress Disorders, Post-Traumatic/ (47535)

72     mental illness.mp. (36376)

73     financial stress.mp. or Financial Stress/ (1786)

74     31 or 32 or 33 or 34 or 35 or 36 or 37 or 38 or 39 or 40 or 41 or 42 or 43 or 44 or 45 or 46 or 47 or 48 or 49 or 50 or 51 or 52 or 53 or 54 or 55 or 56 or 57 or 58 or 59 or 60 or 61 or 62 or 63 or 64 or 65 or 66 or 67 or 68 or 69 or 70 or 71 or 72 or 73 (1709914)

75     covid-19.mp. or COVID-19/ (338566)

76     covid.mp. (340370)

77     coronavirus.mp. or Coronavirus/ (157447)

78     covid influenza.mp. (4)

79     lockdown.mp. (16666)

80     social distance.mp. (1966)

81     social distancing.mp. (9190)

82     Physical Distancing/ or physical distanc*.mp. (5497)

83     omicron.mp. (6594)

84     long covid.mp. (2993)

85     stay-at-home orders.mp. (900)

86     quarantine.mp. or Quarantine/ (14724)

87     pandemic*.mp. (228769)

88     75 or 76 or 77 or 78 or 79 or 80 or 81 or 82 or 83 or 84 or 85 or 86 or 87 (406130)

89     30 and 74 and 88 (1844)

90     limit 89 to yr="2019 -Current" (1801)

***************************

**Database: Embase Classic+Embase**
**Search Strategy:**
**1**  poverty.mp. or Poverty/ (72725)
**2**  economic deprivation.mp. (1196)
**3**  resource scarcity.mp. (714)
**4**  impoverished.mp. (4917)
**5**  penniless.mp. (8)
**6**  inadequate resources.mp. (640)
**7**  poor living conditions.mp. (515)
**8**  inadequate living conditions.mp. (27)
**9**  lack of income.mp. (92)
**10**  no income.mp. (321)
**11**  lack of money.mp. (566)
**12**  no money.mp. (224)
**13**  unemploy*.mp. (40100)
**14**  low ses.mp. (4951)
**15**  low socioeconomic status.mp. or Low Socioeconomic Status/ (9317)
**16**  economic insufficiency.mp. (18)
**17**  Bankruptcy/ or bankrupt*.mp. (126133)
**18**  poor community.mp. (379)
**19**  poor family.mp. (836)
**20**  poor individual*.mp. (241)
**21**  slum*.mp. (5822)
**22**  low income.mp. (67527)
**23**  income insecurity.mp. (51)
**24**  income security.mp. (368)
**25**  financial hardship.mp. (1774)
**26**  equity.mp. (43337)
**27**  health equity.mp. or Health Equity/ (14405)
**28**  inequity.mp. (7722)
**29**  inequitable.mp. (3066)
**30**  financial stress.mp. or Financial Stress/ (3343)
**31**  1 or 2 or 3 or 4 or 5 or 6 or 7 or 8 or 9 or 10 or 11 or 12 or 13 or 14 or 15 or 16 or 17 or 18 or 19 or 20 or 21 or 22 or 23 or 24 or 25 or 26 or 27 or 28 or 29 or 30 (359355)
**32**  mental wellness.mp. (389)
**33**  mental illness.mp. (50846)
**34**  mental stability.mp. (163)
**35**  mental hygiene.mp. (2087)
**36**  social anxiety.mp. (10677)
**37**  life statisfaction.mp. (1)
**38**  psychological health.mp. (10412)
**39**  psychological wellbeing.mp. (4529)
**40**  emotional intelligence.mp. or Emotional Intelligence/ (4859)
**41**  emotional regulation.mp. or Emotional Regulation/ (8543)
**42**  psychological resilience.mp. or Resilience, Psychological/ (9993)
**43**  psychological adaptation.mp. or Adaptation, Psychological/ (3333)
**44**  coping behaviour*.mp. (915)
**45**  mental fatigue.mp. or Mental Fatigue/ (2584)
**46**  alcoholism.mp. or Alcoholism/ (157912)
**47**  psychological stress.mp. or Stress, Psychological/ (96558)
**48**  optimism.mp. or Optimism/ (15599)
**49**  hopeful*.mp. (23867)
**50**  social exclusion.mp. or Social Isolation/ (38144)
**51**  social deprivation.mp. or Social Deprivation/ (35089)
**52**  mood.mp. (180370)
**53**  mood disorder.mp. or Mood Disorders/ (58965)
**54**  affective disorder.mp. (12332)
**55**  psychotic disorder.mp. or Psychotic Disorders/ (64216)
**56**  Schizophrenia/ or schizophrenia.mp. (252857)
**57**  psychosis.mp. (168363)
**58**  substance-use.mp. (70209)
**59**  substance-related disorders.mp. or Substance-Related Disorders/ (42042)
**60**  substance misuse.mp. (5090)
**61**  addiction.mp. (162448)
**62**  personality disorders.mp. or Personality Disorders/ (27326)
**63**  panic disorder.mp. or Panic Disorder/ (21760)
**64**  stress-related disorder.mp. (230)
**65**  trauma-related disorder.mp. (63)
**66**  post traumatic stress disorder.mp. or Stress Disorders, Post-Traumatic/ (56215)
**67**  agoraphobia.mp. or Agoraphobia/ (7747)
**68**  covid-19.mp. or COVID-19/ (418233)
**69**  Covid.mp. (404658)
**70**  coronavirus.mp. or Coronavirus/ (438013)
**71**  covid influenza.mp. (10)
**72**  lockdown.mp. (23165)
**73**  social distance.mp. (6102)
**74**  social distancing.mp. (17486)
**75**  Physical Distancing/ or physical distanc*.mp. (12371)
**76**  omicron.mp. (10176)
**77**  long covid.mp. (6176)
**78**  stay-at-home orders.mp. (989)
**79**  quarantine.mp. or Quarantine/ (21393)
**80**  health emergency.mp. (7388)
**81**  depression/ or depression.mp. (880712)
**82**  mental health/ (211760)
**83**  mental disorder.mp. (16744)
**84**  anxiety/ or anxiety disorder/ (381272)
**85**  covid pandemic.mp. (3490)
**86**  32 or 33 or 34 or 35 or 36 or 37 or 38 or 39 or 40 or 41 or 42 or 43 or 44 or 45 or 46 or 47 or 48 or 49 or 50 or 51 or 52 or 53 or 54 or 55 or 56 or 57 or 58 or 59 or 60 or 61 or 62 or 63 or 64 or 65 or 66 or 67 or 81 or 82 or 83 or 84 (2027799)
**87**  67 or 68 or 69 or 70 or 71 or 72 or 73 or 74 or 75 or 76 or 77 or 78 or 79 or 80 or 85 (514384)
**88**  31 and 86 and 87 (3632)
**89**  limit 88 to yr="2019 -Current" (3501)

**CINAHL**

Poverty OR “economic deprivation” OR “resource scarcity” OR impoverish* OR penniless OR “inadequate resources” OR “poor living conditions” OR “inadequate living conditions” OR “lack of income” OR “no income” OR “lack of money” OR “no money” OR unemployed OR “low ses” OR “low socioeconomic status” OR “economic insufficiency” OR bankrupt* OR “poor community” OR “poor family” OR “poor individual” OR slum* OR “low income” OR “income insecurity” OR “income security” OR “financial hardship” OR equity OR “health equity” OR inequity OR inequitable OR “financial stress”

AND

“Mental health” OR “mental wellness” OR “mental disorder” OR “mental stability” OR “mental hygiene” OR “anxiety disorder” OR “social anxiety” OR anxiety OR “life satisfaction” OR “psychological health” OR “psychological well-being” OR “emotional intelligence” OR “emotional regulation” OR “psychological resilience” OR “psychological adaptation” OR “coping behaviour” OR “mental fatigue” OR depress* OR alcoholism OR “psychological stress” OR optimism OR hopeful OR “social isolation” OR “social exclusion” OR “social deprivation” OR mood OR “mood disorder” OR “affective disorder” OR “psychotic disorders” OR schizophrenia OR psychosis OR “substance use” OR “substance-related disorders” OR “substance misuse” OR addiction OR “personality disorders” OR “panic disorder” OR “stress-related disorders” OR “trauma-related disorders” OR PTSD OR “post-traumatic stress disorder” OR agoraphobia

AND

Covid-19 OR Covid OR coronavirus OR “covid influenza” OR lockdown OR “social distance” OR “social distancing” OR “physical distancing” OR omicron OR “long covid” OR “stay-at-home orders” OR quarantine OR pandemic* OR “health emergency”

**Database: APA PsycInfo**
**Search Strategy:**
**1**  poverty.mp. or Poverty/ (35687)
**2**  economic deprivation.mp. (603)
**3**  resource scarcity.mp. (391)
**4**  impoverished.mp. (3935)
**5**  penniless.mp. (10)
**6**  inadequate resources.mp. (258)
**7**  poor living conditions.mp. (116)
**8**  inadequate living conditions.mp. (9)
**9**  lack of income.mp. (45)
**10**  no income.mp. (95)
**11**  lack of money.mp. (165)
**12**  no money.mp. (88)
**13**  unemploy*.mp. (18405)
**14**  low ses.mp. (3975)
**15**  low socioeconomic status.mp. or Low Socioeconomic Status/ (3774)
**16**  economic insufficiency.mp. (11)
**17**  Bankruptcy/ or bankrupt*.mp. (826)
**18**  poor community.mp. (150)
**19**  poor family.mp. (568)
**20**  poor individual*.mp. (138)
**21**  slum*.mp. (1446)
**22**  low income.mp. (25539)
**23**  income insecurity.mp. (28)
**24**  income security.mp. (153)
**25**  financial hardship.mp. (645)
**26**  equity.mp. (20420)
**27**  health equity.mp. or Health Equity/ (1918)
**28**  inequity.mp. (3602)
**29**  inequitable.mp. (1578)
**30**  financial stress.mp. or Financial Stress/ (4992)
**31**  1 or 2 or 3 or 4 or 5 or 6 or 7 or 8 or 9 or 10 or 11 or 12 or 13 or 14 or 15 or 16 or 17 or 18 or 19 or 20 or 21 or 22 or 23 or 24 or 25 or 26 or 27 or 28 or 29 or 30 (110733)
**32**  mental health.mp. or Mental Health/ (281677)
**33**  mental wellness.mp. (335)
**34**  mental illness.mp. (51370)
**35**  Mental Disorders/ or mental disorder*.mp. (197655)
**36**  mental stability.mp. (123)
**37**  mental hygiene.mp. (4450)
**38**  anxiety disorder.mp. or Anxiety Disorders/ (39626)
**39**  social anxiety.mp. (12506)
**40**  Anxiety/ or anxiety.mp. (285487)
**41**  life statisfaction.mp. (1)
**42**  psychological health.mp. (7952)
**43**  psychological wellbeing.mp. (2246)
**44**  emotional intelligence.mp. or Emotional Intelligence/ (9830)
**45**  emotional regulation.mp. or Emotional Regulation/ (17975)
**46**  psychological resilience.mp. or Resilience, Psychological/ (21505)
**47**  psychological adaptation.mp. or Adaptation, Psychological/ (1163)
**48**  coping behaviour*.mp. (508)
**49**  mental fatigue.mp. or Mental Fatigue/ (1348)
**50**  Depression/ or depress*.mp. (430460)
**51**  alcoholism.mp. or Alcoholism/ (54295)
**52**  psychological stress.mp. or Stress, Psychological/ (13038)
**53**  optimism.mp. or Optimism/ (13074)
**54**  hopeful*.mp. (7696)
**55**  social exclusion.mp. or Social Isolation/ (12565)
**56**  social deprivation.mp. or Social Deprivation/ (1648)
**57**  mood.mp. (95248)
**58**  mood disorder.mp. or Mood Disorders/ (20170)
**59**  affective disorder.mp. (8740)
**60**  psychotic disorder.mp. or Psychotic Disorders/ (35481)
**61**  Schizophrenia/ or schizophrenia.mp. (144912)
**62**  psychosis.mp. (60909)
**63**  substance-use.mp. (74874)
**64**  substance-related disorders.mp. or Substance-Related Disorders/ (40053)
**65**  substance misuse.mp. (3854)
**66**  addiction.mp. (60847)
**67**  personality disorders.mp. or Personality Disorders/ (32814)
**68**  panic disorder.mp. or Panic Disorder/ (13104)
**69**  stress-related disorder.mp. (96)
**70**  trauma-related disorder.mp. (46)
**71**  PTSD.mp. (44850)
**72**  post traumatic stress disorder.mp. or Stress Disorders, Post-Traumatic/ (14400)
**73**  agoraphobia.mp. or Agoraphobia/ (5945)
**74**  covid-19.mp. or COVID-19/ (33505)
**75**  Covid.mp. (33722)
**76**  coronavirus.mp. or Coronavirus/ (10652)
**77**  covid influenza.mp. (0)
**78**  lockdown.mp. (3793)
**79**  social distance.mp. (4759)
**80**  social distancing.mp. (2386)
**81**  Physical Distancing/ or physical distanc*.mp. (2289)
**82**  omicron.mp. (62)
**83**  long covid.mp. (184)
**84**  stay-at-home orders.mp. (338)
**85**  quarantine.mp. or Quarantine/ (2325)
**86**  health emergency.mp. (738)
**87**  covid pandemic.mp. (285)
**88**  32 or 33 or 34 or 35 or 36 or 37 or 38 or 39 or 40 or 41 or 42 or 43 or 44 or 45 or 46 or 47 or 48 or 49 or 50 or 51 or 52 or 53 or 54 or 55 or 56 or 57 or 58 or 59 or 60 or 61 or 62 or 63 or 64 or 65 or 66 or 67 or 68 or 69 or 70 or 71 or 72 or 73 (1246271)
**89**  74 or 75 or 76 or 77 or 78 or 79 or 80 or 81 or 82 or 83 or 84 or 85 or 86 or 87 (40475)
**90**  31 and 88 and 89 (921)
**91**  limit 90 to yr="2019 -Current" (881)

**Nursing and Allied Database**

Poverty OR “economic deprivation” OR “resource scarcity” OR impoverish* OR penniless OR “inadequate resources” OR “poor living conditions” OR “inadequate living conditions” OR “lack of income” OR “no income” OR “lack of money” OR “no money” OR unemployed OR “low ses” OR “low socioeconomic status” OR “economic insufficiency” OR bankrupt* OR “poor community” OR “poor family” OR “poor individual” OR slum* OR “low income” OR “income insecurity” OR “income security” OR “financial hardship” OR equity OR “health equity” OR inequity OR inequitable OR “financial stress”

AND

“Mental health” OR “mental wellness” OR “mental disorder” OR “mental stability” OR “mental hygiene” OR “anxiety disorder” OR “social anxiety” OR anxiety OR “life satisfaction” OR “psychological health” OR “psychological well-being” OR “emotional intelligence” OR “emotional regulation” OR “psychological resilience” OR “psychological adaptation” OR “coping behaviour” OR “mental fatigue” OR depress* OR alcoholism OR “psychological stress” OR optimism OR hopeful OR “social isolation” OR “social exclusion” OR “social deprivation” OR mood OR “mood disorder” OR “affective disorder” OR “psychotic disorders” OR schizophrenia OR psychosis OR “substance use” OR “substance-related disorders” OR “substance misuse” OR addiction OR “personality disorders” OR “panic disorder” OR “stress-related disorders” OR “trauma-related disorders” OR PTSD OR “post-traumatic stress disorder” OR agoraphobia

AND

Covid-19 OR Covid OR coronavirus OR “covid influenza” OR lockdown OR “social distance” OR “social distancing” OR “physical distancing” OR omicron OR “long covid” OR “stay-at-home orders” OR quarantine OR pandemic* OR “health emergency”

**Sociology Abstracts**

Poverty OR “economic deprivation” OR “resource scarcity” OR impoverish* OR penniless OR “inadequate resources” OR “poor living conditions” OR “inadequate living conditions” OR “lack of income” OR “no income” OR “lack of money” OR “no money” OR unemployed OR “low ses” OR “low socioeconomic status” OR “economic insufficiency” OR bankrupt* OR “poor community” OR “poor family” OR “poor individual” OR slum* OR “low income” OR “income insecurity” OR “income security” OR “financial hardship” OR equity OR “health equity” OR inequity OR inequitable OR “financial stress”

AND

“Mental health” OR “mental wellness” OR “mental disorder” OR “mental stability” OR “mental hygiene” OR “anxiety disorder” OR “social anxiety” OR anxiety OR “life satisfaction” OR “psychological health” OR “psychological well-being” OR “emotional intelligence” OR “emotional regulation” OR “psychological resilience” OR “psychological adaptation” OR “coping behaviour” OR “mental fatigue” OR depress* OR alcoholism OR “psychological stress” OR optimism OR hopeful OR “social isolation” OR “social exclusion” OR “social deprivation” OR mood OR “mood disorder” OR “affective disorder” OR “psychotic disorders” OR schizophrenia OR psychosis OR “substance use” OR “substance-related disorders” OR “substance misuse” OR addiction OR “personality disorders” OR “panic disorder” OR “stress-related disorders” OR “trauma-related disorders” OR PTSD OR “post-traumatic stress disorder” OR agoraphobia

AND

Covid-19 OR Covid OR coronavirus OR “covid influenza” OR lockdown OR “social distance” OR “social distancing” OR “physical distancing” OR omicron OR “long covid” OR “stay-at-home orders” OR quarantine OR pandemic* OR “health emergency”
