# Supplementary material for: Experiences of mental health and poverty in high-income countries during COVID-19: A systematic review and meta-aggregation
Source: PLOS Ment Health. 2024 Oct 21;1(5):e0000059. doi: 10.1371/journal.pmen.0000059 (PMC12798167; doi:10.1371/journal.pmen.0000059)
Supplement: S1 File — (DOCX) [file pmen.0000059.s003.docx]

**Excluded Articles and Reasons for Exclusion**

**#1222 - Spencer 2022**

Women's Lived Experiences with Temporary Assistance for Needy Families (TANF): How TANF Can Better Support Women's Wellbeing and Reduce Intimate Partner Violence.

Spencer, Rachael A; Lemon, Emily D; Komro, Kelli A; Livingston, Melvin D; Woods-Jaeger, Briana

International journal of environmental research and public health / 2022;19(3):

Switzerland 2022 /

DOI: 10.3390/ijerph19031170 · Ref ID: 35162193

Date excluded: 31/07/23

Reason for Exclusion: Not focused on mental wellbeing

**#1586 - Rothe 2021**

Structural Inequities and the Impact of COVID-19 on Latinx Children: Implications for Child and Adolescent Mental Health Practice.

Rothe, Eugenio M; Fortuna, Lisa R; Tobon, Amalia Londono; Postlethwaite, Alejandra; Sanchez-Lacay, J Arturo; Anglero, Yohanis Leonor

Journal of the American Academy of Child and Adolescent Psychiatry / 2021;60(6):669-671

United States 2021 /

DOI: 10.1016/j.jaac.2021.02.013 · Ref ID: 33662497

Date Excluded: 28/07/23

Reason for Exclusion: Wrong study design

**#5219 - Dooley 2020**

Low-Income Children and Coronavirus Disease 2019 (COVID-19) in the US

Dooley D.G.; Bandealy A.; Tschudy M.M.

JAMA Pediatrics / 2020;174(10):924-925

United States American Medical Association 2020 /

DOI: 10.1001/jamapediatrics.2020.2065 · Ref ID: 631867325

Date Excluded: 28/07/23

Reason for Exclusion: Wrong study design

**#1902 - Kawohl 2020**

COVID-19, unemployment, and suicide.

Kawohl, Wolfram; Nordt, Carlos

The lancet. Psychiatry / 2020;7(5):389-390

England 2020 /

DOI: 10.1016/S2215-0366(20)30141-3 · Ref ID: 32353269

Date Excluded: 28/07/23

Reason for Exclusion: Wrong study design

**#4073 - Shaligram 2021**

Equity and Social Justice Concerns in Mental Health Risk during the Pandemic

Shaligram D.; Okpaku S.; Sorel E.

Canadian Journal of Psychiatry / 2021;66(12):1099-1100

Canada SAGE Publications Inc. 2021 /

DOI: 10.1177/07067437211027979 · Ref ID: 2012906559

Date Excluded: 28/07/23

Reason for Exclusion: Wrong study design

**#758 - Adegboye 2021**

Understanding why the COVID-19 pandemic-related lockdown increases mental health difficulties in vulnerable young children.

Adegboye, Dolapo; Williams, Ffion; Collishaw, Stephan; Shelton, Katherine; Langley, Kate; Hobson, Christopher; Burley, Daniel; van Goozen, Stephanie

JCPP advances / 2021;1(1):e12005

United States 2021 /

DOI: 10.1111/jcv2.12005 · Ref ID: 34485985

Date Excluded: 27/07/23

Reason for Exclusion: Wrong study design

**#5380 - Tandon 2020**

COVID-19 and mental health: Preserving humanity, maintaining sanity, and promoting health

Tandon R.

Asian Journal of Psychiatry / 2020;51((Tandon) Department of Psychiatry, WMU Homer Stryker School of Medicine, Kalamazoo, MI, United States):102256

Netherlands Elsevier B.V. 2020 /

DOI: 10.1016/j.ajp.2020.102256 · Ref ID: 2006829325

Date Excluded: 28/07/23

Reason for Exclusion: Wrong study design

**#2794 - Hertz-Palmor 2021**

Association among income loss, financial strain and depressive symptoms during COVID-19: Evidence from two longitudinal studies

Hertz-Palmor N.; Moore T.M.; Gothelf D.; DiDomenico G.E.; Dekel I.; Greenberg D.M.; Brown L.A.; Matalon N.; Visoki E.; White L.K.; Himes M.M.; Schwartz-Lifshitz M.; Gross R.; Gur R.C.; Gur R.E.; Pessach I.M.; Barzilay R.

Journal of affective disorders / 2021;291((Hertz-Palmor) Sheba Medical Center, Ramat Gan, Israel; School of Psychological Sciences, Tel Aviv University, Tel Aviv, Israel(Moore, Gur) University of Pennsylvania Perelman School of Medicine, Department of Psychiatry, Philadelphia, PA, USA; Lifespan B):1-8

Netherlands NLM (Medline) 2021 /

DOI: 10.1016/j.jad.2021.04.054 · Ref ID: 635147799

Date Excluded: 28/07/23

Reason for Exclusion: Wrong study design

**#3889 - Fiocco 2021**

Stress and adjustment during the covid-19 pandemic: A qualitative study on the lived experience of canadian older adults

Fiocco A.J.; Gryspeerdt C.; Franco G.

International Journal of Environmental Research and Public Health / 2021;18(24):12922

Switzerland MDPI 2021 /

DOI: 10.3390/ijerph182412922 · Ref ID: 2014780944

Date Excluded: 27/07/23

Reason for Exclusion: Not focused on low-income participants

**#364 - Jenkins 2023**

Depression and anxiety among multiethnic middle school students: Age, gender, and sociocultural environment.

Jenkins, Janis H; Sanchez, Giselle; Miller, Eric A; Santillanes Allande, Nadia Irina; Urano, Grace; Pryor, Alexandra J

The International journal of social psychiatry / 2023;69(3):784-794

England 2023 /

DOI: 10.1177/00207640221140282 · Ref ID: 36529994

Date Excluded: 31/07/23

Reason for Exclusion: Not adequate qualitative findings

**#907 - Purtle 2020**

COVID-19 and mental health equity in the United States.

Purtle, Jonathan

Social psychiatry and psychiatric epidemiology / 2020;55(8):969-971

Germany 2020 /

DOI: 10.1007/s00127-020-01896-8 · Ref ID: 32556376

Date Excluded: 27/07/23

Reason for Exclusion: Wrong study design

**#930 - Boyers 2022**

Period poverty: The perceptions and experiences of impoverished women living in an inner-city area of Northwest England.

Boyers, Madeleine; Garikipati, Supriya; Biggane, Alice; Douglas, Elizabeth; Hawkes, Nicola; Kiely, Ciara; Giddings, Cheryl; Kelly, Julie; Exley, Diane; Phillips-Howard, Penelope A; Mason, Linda

PloS one / 2022;17(7):e0269341

United States 2022 /

DOI: 10.1371/journal.pone.0269341 · Ref ID: 35834506

Date Excluded: 31/07/23

Reason for Exclusion: Not focused on mental wellbeing

**#1816 - Silverman 2020**

Early pregnancy mood before and during COVID-19 community restrictions among women of low socioeconomic status in New York City: a preliminary study.

Silverman, Michael E; Medeiros, Cathryn; Burgos, Laudy

Archives of women's mental health / 2020;23(6):779-782

Austria 2020 /

DOI: 10.1007/s00737-020-01061-9 · Ref ID: 32844329

Date Excluded: 27/07/23

Reason for Exclusion: Wrong study design

**#5209 - Kavoor 2020**

COVID-19 in People with Mental Illness: Challenges and Vulnerabilities

Kavoor A.R.

Asian Journal of Psychiatry / 2020;51((Kavoor) Central Queensland Mental Health Alcohol and Other Drugs Services, Queensland, Australia and Faculty of Medicine, University of Queensland, Australia):102051

Netherlands Elsevier B.V. 2020 /

DOI: 10.1016/j.ajp.2020.102051 · Ref ID: 2005547382

Date Excluded: 27/07/23

Reason for Exclusion: Wrong study design

**#1671 - Bateman 2021**

Confronting COVID-19 in under-resourced, African American neighborhoods: a qualitative study examining community member and stakeholders' perceptions.

Bateman, Lori Brand; Schoenberger, Yu-Mei M; Hansen, Barbara; Osborne, Tiffany N; Okoro, Grace C; Speights, Kimberly M; Fouad, Mona N

Ethnicity & health / 2021;26(1):49-67

England 2021 /

DOI: 10.1080/13557858.2021.1873250 · Ref ID: 33472411

Date Excluded: 27/07/23

Reason for Exclusion: Not focused on mental wellbeing

**#8394 - Flores-Flores 2020**

“We can’t carry the weight of the whole world”: illness experiences among Peruvian older adults with symptoms of depression and anxiety

Flores-Flores, Oscar; Zevallos-Morales, Alejandro; Carrión, Ivonne; Pawer, Dalia; Rey, Lorena; Checkley, W.; Hurst, J. R.; Siddharthan, T.; Parodi, Jose F.; Gallo, Joseph J.; Pollard, Suzanne L.

International Journal of Mental Health Systems 2020;14(1):49

2020

DOI: 10.1186/s13033-020-00381-8 · Ref ID: Flores-Flores2020

Date Excluded: 07/09/23

Reason for Exclusion: No reference to COVID19

**#4487 - Whitehead 2021**

Poverty, health, and covid-19

Whitehead M.; Taylor-Robinson D.; Barr B.

The BMJ / 2021;372((Whitehead, Taylor-Robinson, Barr) Department of Public Health Policy and Systems, University of Liverpool, Liverpool, United Kingdom):372

United Kingdom BMJ Publishing Group 2021 /

DOI: 10.1136/bmj.n376 · Ref ID: 634193585

Date Excluded: 27/07/23

Reason for Exclusion: Wrong study design

**#1959 - Mahajan 2021**

COVID-19 and its socioeconomic impact

Mahajan A.

Cancer Research, Statistics, and Treatment / 2021;4(1):12-18

India Wolters Kluwer Medknow Publications 2021 /

DOI: 10.4103/crst.crst_29_21 · Ref ID: 636638885

Date Excluded: 27/07/23

Reason for Exclusion: Wrong study design

**#115 - Hibel 2021**

The psychological and economic toll of the COVID-19 pandemic on Latina mothers in primarily low-income essential worker families.

Hibel, Leah C; Boyer, Chase J; Buhler-Wassmann, Andrea C; Shaw, Blake J

Traumatology / 2021;27(1):40-47

England 2021 /

DOI: 10.1037/trm0000293 · Ref ID: 37383674

Date Excluded: 27/07/23

Reason for Exclusion: Wrong study design

**#5787 - Childress 2023**

Exploring the lived experiences of women with children during COVID-19: Maternal stress and coping mechanisms.

Childress, Saltanat; Roberts, Alison; LaBrenz, Catherine A.; Findley, Erin; Ekueku, Modesty; Baiden, Philip

Children & Youth Services Review 02// 2023;145():N.PAG-N.PAG

Pergamon Press - An Imprint of Elsevier Science 2023 02//

DOI: 10.1016/j.childyouth.2022.106775 · Ref ID: 161442564

Date Excluded: 27/07/23

Reason for Exclusion: Not focused on low-income participants

**#688 - May 2021**

Socioeconomic and Psychosocial Adversities Experienced by Freelancers Working in the UK Cultural Sector During the COVID-19 Pandemic: A Qualitative Study.

May, Tom; Warran, Katey; Burton, Alexandra; Fancourt, Daisy

Frontiers in psychology / 2021;12(101550902):672694

Switzerland 2021 /

DOI: 10.3389/fpsyg.2021.672694 · Ref ID: 35145444

Date Excluded: 31/07/23

Reason for Exclusion: Not focused on low-income participants

**#6095 - Ward 2020**

Money woes and distance: Patient stressors during the COVID-19 era.

Ward, Brian

Medical Environment Update 09// 2020;30(9):1-3

Danvers, Massachusetts HCPro 2020 09//

Ref ID: 145185281

Date Excluded: 27/07/23

Reason for Exclusion: Wrong study design

**#712 - Lu 2021**

COVID-19, Economic Impact, Mental Health, and Coping Behaviors: A Conceptual Framework and Future Research Directions.

Lu, Xiaoqian; Lin, Zhibin

Frontiers in psychology / 2021;12(101550902):759974

Switzerland 2021 /

DOI: 10.3389/fpsyg.2021.759974 · Ref ID: 34899503

Date Excluded: 31/07/23

Reason for Exclusion: Wrong study design

**#1500 - Parra-Saavedra 2021**

Maternal mental health is being affected by poverty and COVID-19.

Parra-Saavedra, Miguel; Miranda, Jezid

The Lancet. Global health / 2021;9(8):e1031-e1032

England 2021 /

DOI: 10.1016/S2214-109X(21)00245-X · Ref ID: 34175005

Date Excluded: 27/07/23

Reason for Exclusion: Wrong study design

**#1317 - Amran 2022**

Psychosocial risk factors associated with mental health of adolescents amidst the COVID-19 pandemic outbreak.

Amran, Muhammad Syawal

The International journal of social psychiatry / 2022;68(1):6-8

England 2022 /

DOI: 10.1177/0020764020971008 · Ref ID: 33158391

Date Excluded: 31/07/23

Reason for Exclusion: Not focused on low-income participants

**#1065 - Leitao 2022**

Perspectives and experiences of Covid-19: Two Irish studies of families in disadvantaged communities.

Leitao, Catarina; Shumba, Jefrey; Quinn, Marian

PloS one / 2022;17(7):e0270472

United States 2022 /

DOI: 10.1371/journal.pone.0270472 · Ref ID: 35849569

Date Excluded: 31/07/23

Reason for Exclusion: Wrong study design

**#347 - Barwise 2023**

Community engaged research to measure the impact of COVID-19 on vulnerable community member's well-being and health : A mixed methods approach.

Barwise, Amelia K; Egginton, Jason; Pacheco-Spann, Laura; Clift, Kristin; Albertie, Monica; Johnson, Matthew; Batbold, Sarah; Phelan, Sean; Allyse, Megan

Wiener klinische Wochenschrift / 2023;135(9-10):221-227

Austria 2023 /

DOI: 10.1007/s00508-022-02113-z · Ref ID: 36469122

Date Excluded: 31/07/23

Reason for Exclusion: Wrong study design

**#5702 - Vicerra 2022**

Mental stress and well-being among low-income older adults during COVID-19 pandemic.

Vicerra, Paolo

Asian Journal of Social Health & Behavior 07//Jul-Sep2022 2022;5(3):101-107

Wolters Kluwer India Pvt Ltd 2022 07//Jul-Sep2022

DOI: 10.4103/shb.shb_110_22 · Ref ID: 158678527

Date Excluded: 27/07/23

Reason for Exclusion: Wrong study design

**#6341 - Feng 2021**

Evaluation and analysis of mental health level of college students with financial difficulties under the background of COVID-19.

Feng, Yongpeng; Zhang, Yunting

Frontiers in Psychology / 2021;12(Chen, G. (2008). Discussion on mental health education of impoverished college students from the perspective of psychological capital. China Adult Educ.38-39.Dong, X., Liu, J., Wang, R., & Song, L. (2016). Study on mental health status of students with fi):

Switzerland Frontiers Media S.A.Switzerland 2021 /

DOI: 10.3389/fpsyg.2021.649195 · Ref ID: 2021-40142-001

Date Excluded: 31/07/23

Reason for Exclusion: Not focused on low-income participants

**#3114 - Goh 2022**

Why did COVID-19 not further harm the mental health of poor mothers? A mixed-method study on low-income families in Singapore

Goh E.C.L.; Wen D.J.R.; Ang R.C.Y.

BMJ open / 2022;12(1):e052103

United Kingdom NLM (Medline) 2022 /

DOI: 10.1136/bmjopen-2021-052103 · Ref ID: 637122110

Date Excluded: 31/07/23

Reason for Exclusion: Not adequate qualitative findings

**#156 - Williams 2023**

COVID-19 stressors for Hispanic/Latino patients living with type 2 diabetes: a qualitative study.

Williams, Myia S; Cigaran, Edgardo; Martinez, Sabrina; Marino, Jose; Barbero, Paulina; Myers, Alyson K; DiClemente, Ralph J; Goris, Nicole; Gomez, Valeria Correa; Granville, Dilcia; Guzman, Josephine; Harris, Yael T; Kline, Myriam; Lesser, Martin L; Makaryus, Amgad N; Murray, Lawrence M; McFarlane, Samy I; Patel, Vidhi H; Polo, Jennifer; Zeltser, Roman; Pekmezaris, Renee

Frontiers in clinical diabetes and healthcare / 2023;4(9918266295306676):1070547

Switzerland 2023 /

DOI: 10.3389/fcdhc.2023.1070547 · Ref ID: 37187937

Date Excluded: 31/07/23

Reason for Exclusion: Not focused on low-income participants

© 2024 Covidence
